# PEIMAN 1.0: Post-translational modification Enrichment, Integration and Matching ANalysis

**DOI:** 10.1093/database/bav037

**Published:** 2015-04-23

**Authors:** Payman Nickchi, Mohieddin Jafari, Shiva Kalantari

**Affiliations:** ^1^Protein Chemistry & Proteomics Unit, Biotechnology Research Center, Pasteur Institute of Iran, 69, Pasteur St., 13164 Tehran, Iran, ^2^School of Biological Sciences, Institute for Research in Fundamental Sciences (IPM), P. O. Box 193955746, Tehran, Iran and ^3^Chronic Kidney Disease Research Center (CKDRC), Shahid Beheshti University of Medical Sciences, Tehran, Iran

## Abstract

Conventional proteomics has discovered a wide gap between protein sequences and biological functions. The third generation of proteomics was provoked to bridge this gap. Targeted and untargeted post-translational modification (PTM) studies are the most important parts of today’s proteomics. Considering the expensive and time-consuming nature of experimental methods, computational methods are developed to study, analyze, predict, count and compute the PTM annotations on proteins. The enrichment analysis softwares are among the common computational biology and bioinformatic software packages. The focus of such softwares is to find the probability of occurrence of the desired biological features in any arbitrary list of genes/proteins. We introduce Post-translational modification Enrichment Integration and Matching Analysis (PEIMAN) software to explore more probable and enriched PTMs on proteins. Here, we also represent the statistics of detected PTM terms used in enrichment analysis in PEIMAN software based on the latest released version of UniProtKB/Swiss-Prot. These results, in addition to giving insight to any given list of proteins, could be useful to design targeted PTM studies for identification and characterization of special chemical groups.

**Database URL:** http://bs.ipm.ir/softwares/PEIMAN/

## Introduction

Any molecular changes on the primary structure of proteins are known as post-translational modifications (PTMs). These modifications, whose count is ∼500, could contain enzymatically or non-enzymatically addition/deletion of chemical groups of on/off amino acids. These evolutionary low-cost alterations which are variable and dynamic affect protein structure as well as protein function (1, 2).

The functional variation of expressed proteins is currently the challenge of conventional first and second generation proteomics (3). PTMs as well as pre- and post-transcriptional regulation of protein expression trigger, terminate, alter different biological processes and also physiological appearance (4). For instance, although over-expression of membrane receptor indicates probable activation of a given signaling pathway, it is not a conclusive evidence. Regardless of regulator loops and crosstalks in signaling networks, many molecular mediators transduce signals by means of PTMs which alter their functionalities (5, 6).

For each PTM types, there have been proposed and experimentally developed targeted and untargeted studies (7–9). These studies become more problematic when the more transient or the more combinatorial PTMs, such as phosphorylation or glycosylation, are considered (2, 10, 11). Computational approaches try to bridge the gap between experimental limitations and what is expected from the PTMs on identified proteins by predicting PTM sites on the sequences (12–19).

Any biological clue which refers to a type of PTMs can certainly help designing a more efficient proteomic study approach. We suggest using enrichment analysis to trigger a popular consideration to PTMs in proteomics. Similar to gene ontology enrichment analysis (20), this analysis is effective in comparing differentially expressed proteins in the sequence of PTM occurrences.

In the context of PTM, some software and databases have been already released. Proteome-wide PTM statistics, proposed by Khoury *et al.*, is a web-based PTM quantifier for curating PTM terms in UniProt. SysPTM 2 is an updated systematic resource for PTM types and holds a module called PTMGO, which is designed for PTM enrichment. PHOSIDA is the first software for predicting post-transnationally modified sites. Its current release can predict phosphorylation and acetylation sites in proteins among five organisms. dbPTM is another PTM-based database providing PTM information. It also provides protein–protein interaction and domain–domain interaction inside the database to determine the functional association of PTM sites located in protein-interacting domains. PEIMAN is a software with a more comprehensive database, especially designed for PTM enrichment analysis. PEIMAN covers more than 500 PTM types and more than 8000 different species which their protein annotations is manually reviewed and exists in UniProt database. The undertaken procedure to create PEIMAN database is demonstrated in the ‘Experimental procedures’ section.

PEIMAN is a standalone software to discover more probable and enriched PTMs. In this software, the latest version of the UniProtKB database was used to extract PTM terms and analysis. The software is freely accessible to be downloaded from http://bs.ipm.ir/softwares/PEIMAN/. The software and its database are supported for 5 years and the database will be updated as the new release of UniProt/SwissProt database is released.

## Experimental procedures

### Preparing the PEIMAN database for enrichment

**Figure 1.**
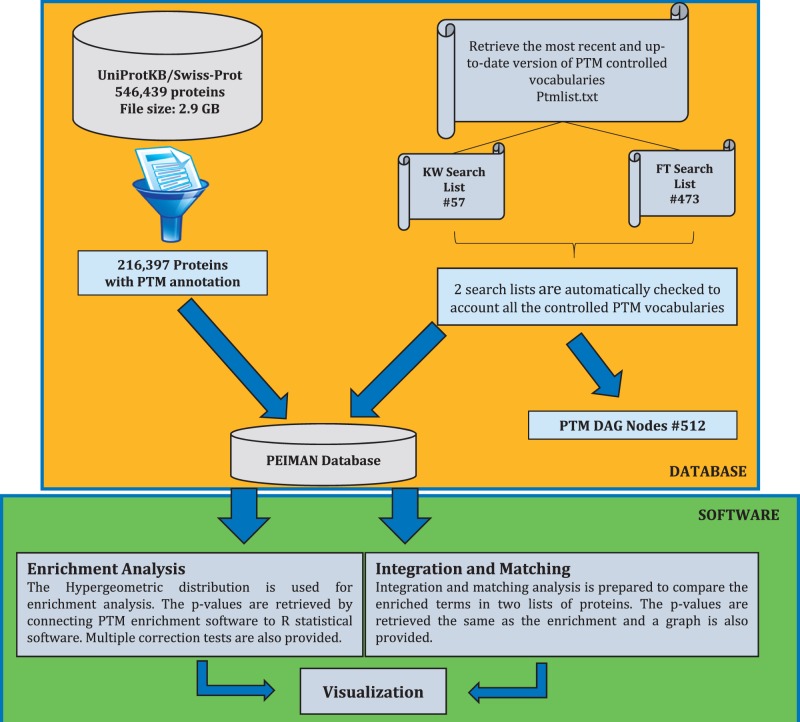
The schematic procedure to exploit PTM information. This figure shows the undertaken procedure to create the ‘*PEIMAN Database’*, necessary for PTM enrichment and visualization. The complete downloaded UniProtKB/Swiss-Prot database, which was manually reviewed and consists of 546 439 proteins (October 2014), was selected. Filtering process just returns back the ID, AC, OS, CC, KW, FT and DR for protein with PTM annotations, necessary for enrichment and save them in ‘216,397 proteins with PTMs annotation’ table. ‘PEIMAN Database’ has all the necessary information about a protein which is required for enrichment and visualization. The total number of proteins with PTM annotation is 216 397.

The procedure for preparing PEIMAN database is demonstrated briefly in Figure 1. As shown, four steps are taken into account to retrieve the desired database as (i) the most recent version (October 2014) of the UniProtKB/Swiss-Prot database was downloaded (21), (ii) the database was filtered and the necessary fields were retrieved, (iii) two search lists were prepared from the most recent version of PTM vocabulary and (iv) the PEIMAN database was created based on the PTM vocabulary. At the first step, UniProtKB website (http://uniprot.org) was used to gather PTM information about proteins. The downloaded DAT file contains 546 439 proteins with size of ∼2.9 GB (ftp://ftp.uniprot.org/pub/databases/uniprot/current_release/knowledgebase/complete/uniprot_sprot.dat.gz).

In the second step, the downloaded file is filtered in order to separate post-translationally modified proteins. Initially, it seems that the PTM vocabulary of each protein is annotated in three fields in DAT files, namely: CC (Comment lines), KW (Keyword lines) and FT (Feature table). However, our assessment showed that the information in CC field is not as accurate and well-curated as KW and FT for PTM annotation. Therefore, we focused on KW and FT fields to search for the controlled PTM vocabularies and the final curation. These two fields are organized differently enabling us to find various levels of information about PTM. The proteins containing PTM annotations are selected out of UniProtKB database (#216 397).

In this step, the values of ID (Identification), AC (Accession number), CC, KW, FT, DR (Database cross-reference) and OS (Organism species) are saved for each protein. The ID field is the identification of each protein containing a UniProtKB internal ID, entry name and the status of each protein that indicates whether it is a reviewed protein (UniProtKB/Swiss-Prot) or not (UniProtKB/TrEMBL). The KW field provides information that can be used to generate indexes of the sequence entries based on functional, structural or other categories. The FT field provides an accurate means for the annotation of the sequence data. These tables also show that regions or sites of interest in the sequence in terms of PTMs, binding sites, enzyme active sites, local secondary structure or other characteristics reported are used as pointers to information in external data resources (21).

**Figure 2.**
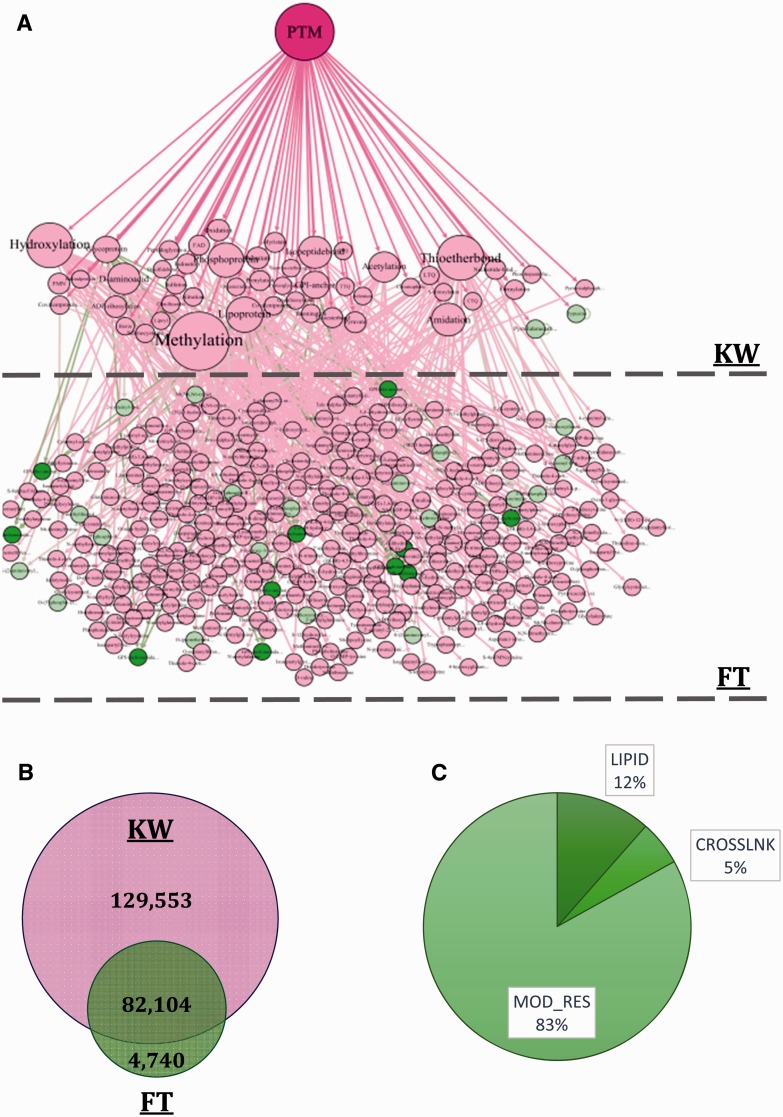
(**A**) The constructed DAG is also depicted in the figure. The DAG consists of three levels: PTM, KW and FT, respectively. Some of the nodes are shown as sample to explicitly demonstrate the DAG structure. For example, d-4-hydroxyvaline and d-valine are both categorized in FT list and are located at the third level of the DAG. Both of these terms are the child of d-amino acid which is categorized in KW list and is located at the second level of the DAG. (**B**) The information about 216 397 proteins which somehow have valuable PTM vocabulary is also depicted. The information about PTM type is saved in one of the two fields: KW or FT. 129 553 (60%) proteins have PTM annotations in KW and 4740 (2%) proteins have the vocabularies just in FT field. Number of proteins that have vocabularies both in KW and FT is 82 104 (38%). (**C**) We consider proteins having three types of PTM annotation namely, LIPID, CROSSLNK and MOD_RES in UniProt database which indicates changes in the structure of proteins. The frequency of proteins having MOD_RES, LIPID and CROSSLNK is 62 675 (83.07%), 8681 (11.53%) and 4091 (5.4%), respectively.

In the third step, the provided information and terms included in KW and FT fields were used to build the PTM Directed Acyclic Graph (DAG) that contains three levels. The first level or root node is ‘PTM’ word at the top of the DAG. The second and the third levels are KW and FT terms, respectively. The PTM DAG was provided as an XML file and is available in the Supplementary File 1. An exemplary PTM DAG and frequency of proteins having PTM annotations in each field of interest are illustrated in Figure 2A and B. In the final step, PTM DAG was applied for gathering post-translationally modified proteins in a definite structure and the PEIMAN database was created. This step is essential for PTM enrichment analysis and visualization.

### Enrichment, integration and matching analysis

PTM enrichment analysis module allows the user to search for enriched terms in a protein list. The enrichment analysis is performed using the hypergeometric statistical test (20). Formally, the hypergeometric distribution is a discrete distribution for finding the probability of *x* successes in *n* draws without replacement from a finite population of size *N* where *K* of them have the desired feature and are labeled as success. The *P*-value for observing *m* proteins having a PTM term can be computed as is shown below:

(1) $$P\text{-value}=\sum_{x=m}^{\min(K,n)}\frac{\left(\begin{array}{l} K\\ x \end{array}\right)\left(\begin{array}{l} N-K\\ n-x \end{array}\right)}{\left(\begin{array}{l} N\\ n \end{array}\right)}.$$

The well-known Bonferroni correction and Benjamini– Hochberg false discovery rate (FDR) test is also included to reduce the effect of multiple testing error. Bonferroni correction is a multiple testing correction method which controls the family-wise error rate (FWER). FWER is the probability of making at least one type I error. In the context of multiple testing corrections, the Bonferroni correction is conservative especially among tests that are not mutually independent. Another approach in multiple testing corrections is to control the FDR which is the expected proportion of identified false positives between positively identified tests. Benjamini–Hochberg FDR is also included in the software to have more power in identifying enriched terms (22). It is obvious in Equation (1) that the calculation of *P*-value largely depends on the calculation of combination of each parameter, that is $\left(\begin{array}{c} N\\ n \end{array}\right)=\frac{N!}{n!(N-n)!}$. In order to make the software more reliable and be as fast as possible in computations, all the necessary calculations for enrichment and retrieving the *P*-values for each PTM term are executed by ‘R statistical software’ and ‘stats package’. PEIMAN software uses an internal connection to R software, passes the parameters to RScript.exe (which was designed to script running in R) and fulfills the calculations. Hence, R statistical software should be installed on the target machine.

In the case of comparative analysis, PEIMAN analyzes two distinct lists of given proteins which is followed by integration enrichment results and matching the significant terms. In other words, two protein lists are investigated for enriched PTM terms and these highly enriched terms of both lists are provided in a table with the corresponding percentage and *P*-values in list 1 and list 2, respectively. A bar plot will also demonstrate the differences of enriched PTM terms in both lists.

The ‘PTM Frequency Analysis’ module provides the frequency analysis of each PTM type which is found in the selected organism and shows the percent and frequency of each type in the given protein in list 1. A bar plot is also provided to better demonstrate the frequency of each type in the organism.

## Results

The ‘216,397 proteins with PTM annotations’ dataset was applied to find the frequency of the PTM terms in each KW and FT field, individually. The frequency of proteins containing PTM information in these fields demonstrated using Venn diagram in Figure 2B. The values for KW, FT and both of the fields were 129 553 (∼60%), 4740 (∼2%) and 82 104 (∼38%), respectively. This figure shows that the frequency of proteins with PTM information in KW field was clearly larger than FT. This means that most of the PTM annotations could be retrieved by searching in KW. However, ∼38% of proteins have the PTM annotations both in KW and FT and ∼2% have PTM annotations only in FT field. Figure 2C shows the frequency of proteins having LIPID, CROSSLNK and MOD_RES inside the FT field. As shown, most of the proteins whose PTM annotations are found in the FT field have MOD_RES inside it.

**Figure 3.**
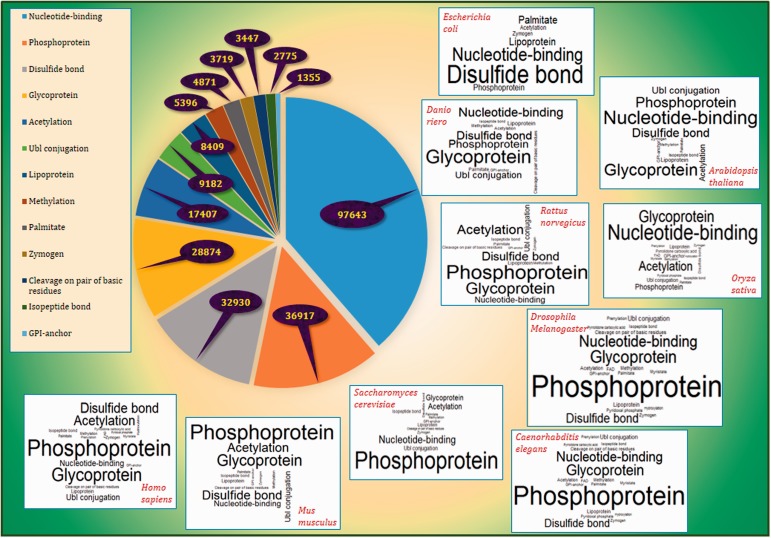
The frequency of PTM terms. The pie plot demonstrates that Nucleotide binding, Phosphoprotein and Disulfide bond have the highest frequency among other PTM vocabularies in UniProtKB/Swiss-Prot with frequencies 97 643, 36 917 and 32 930, respectively. The figure also provides the word clouds of PTM terms in 10 of the well-known model organisms namely *H. sapiens*, *M. musculus*, *R. norvegicus*, *D. melanogaster*, *D. riero* (Zebrafish), *C. elegans*, *S. cerevisiae*, *A. thaliana*, *O. sativa* (Rice) and *E. coli*. More details are presented in Supplementary File 2.

Frequency of PTM annotations in PEIMAN database with a pie-type chart with emphasis on the 10 of the well-known model organisms namely *Homo sapiens*, *Mus musculus*, *Rattus norvegicus*, *Saccharomyces cerevisiae*, *Arabidopsis thaliana*, *Drosophila melanogaster*, *Danio riero* (Zebrafish), *Caenorhabditis elegans*, *Oryza sativa* (Rice) and *Escherichia coli* is illustrated in Figure 3 and Supplementary File 2. The results showed relative similarity between *H. sapiens* and *M. musculus* in terms of PTM frequencies. The order of frequencies in mentioned organisms were as follows: Phosphoprotein, Glycoprotein and Disulfide bond were the top three most viewed terms in comparison to other PTM terms in *H. sapiens* and *M. musculus,* whereas Glycoprotein, Nucleotide-binding and Disulfide bond were the most frequent in *D. riero* (Zebrafish) and *C. elegans*. The Phosphoprotein and Glycoprotein are the most repeated terms in *R. norvegicus* and *D. melanogaster*, but Acetylation and Nucleotide binding are the third most viewed one in each organism, respectively. Nucleotide binding and Glycoprotein were the two top ranked PTM terms in *A. thaliana* and *O. sativa* (Rice) while Phosphoprotein and Disulfide bond were the third most viewed PTM terms in these organisms. Finally, the top three PTM terms for *S. cerevisiae* included Phosphoprotein, Nucleotide binding and Acetylation while disulfide bond, Nucleotide binding and lipoprotein for *E. coli*.

**Figure 4.**
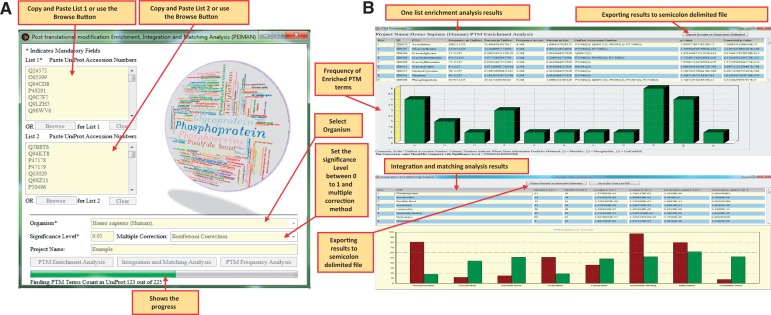
The PEIMAN environment. (**A**) Input parameters in PEIMAN software. The fields with star sign indicates the mandatory input parameters in the software. (**B**) PEIMAN output. The figure demonstrates the output of PTM Enrichment analysis in software. A table and a bar chart are produced after the analysis is completed. The table provides the ID column for each PTM vocabulary. It also provides the frequency of each PTM vocabulary in UniProtKB and the corresponding frequency in protein list. The percent of each PTM vocabulary in UniProtKB and given list of protein is provided as well. The table shows which UniProtKB accession numbers in protein list have the corresponding PTM vocabularies. A *P*-value and corrected *P*-value (if multiple correction method is chosen) are provided for further analysis. A bar chart is provided to better represent the data. A comment about where more information is accessible about each protein (Database cross-reference—DR) is provided as well. The result of the integration and matching analysis are presented for two separate protein lists. The produced table and bar chart gives a better understanding about the PTM vocabularies found in two separate protein lists. The table provides the *P*-value, corrected *P*-value, frequency and percent of each PTM vocabulary in both lists.

Figure 4A presents the input parameters of the software. It is possible to do enrichment analysis for one list of proteins or do this analysis for two separate lists to compare them. It is also possible to perform PTM frequency analysis for list 1. The protein lists could be easily pasted or imported from a text file. The next input module is to select the organism included in all 8685 organisms available in UniProtKB/Swiss-Prot. The ‘significance level’ and ‘multiple testing correction’ were also considered in the software. It is possible to choose any value for significance level and also different modes of corrections; ‘No multiple correction’, ‘Bonferroni Correction’ and ‘Benjamini– Hochberg FDR Test’. The speed of analysis will greatly depends on the counts of PTM vocabularies found in PEIMAN database for the selected organism.

The output for PTM enrichment analysis consisted of a table, a bar chart, some comments at the bottom of the form and a button for exporting results to delimited text file (Figure 4B). The table in the file consisted of ID, PTM vocabularies, frequency in UniProt, percentage in UniProt, frequency in the list, percentage in the list, UniProtKB accession number, *P*-value and corrected *P*-value columns. The ID column is a unique ID which separates the PTM vocabularies in order to be unique in the results and solely used in the software and PTM DAG. In the UniProtKB accession number column, the cross-reference to specific PTM database was also provided. Numbers 1, 2 and 3 in the parenthesis indicate that the cross-reference information could be obtained in PhosSite (23), PhosphoSite (24) or UniCarbKB (25), respectively, for the mentioned protein. The modified significance level is also available at the bottom of the form which applied for comparison with corrected *P*-value when ‘Bonferroni Correction’ multiple correction test is selected. A bar chart was also provided to explain PTM frequencies visually.

The ‘Integration and Matching Analysis’ module, another capability of the software, is also shown in Figure 4B. In this module, enrichment analysis results were provided after integrating and matching the significantly enriched PTM terms of the two lists of proteins. Accordingly, it was possible to extract more information about two lists mutually. Two buttons were also provided at the top of the form to export the bar chart and table to PDF and delimited text files, respectively.

## Discussion

Due to high focus devoted to PTM topic and its importance in protein destination in a cellular systems, mass spectrometry-based proteomic studies were developed to provide tremendous accurate information regarding PTMs (3). Besides the high cost and time-consuming experimental studies, a few computational efforts have been started recently. Lachmann and Ma’ayan (19) introduced the Kinase enrichment analysis which performs enrichment analysis in kinase terms to explore proteins with this enzymatic action and their targets. The PTMcode in two different releases, proposed by Minguez *et al.* (15, 17), focused on prediction of PTMs based on literature survey, co-evolution of the residue, structural proximity and exploring PTM hotspots. However, PEIMAN software focused on three domains about PTMs, namely accounting all known and predicted PTMs, PTM DAG reconstruction and PTM enrichment analysis.

Regards to accounting PTMs, Khoury *et al*. (26) provided a proteome-wide PTM statistics curator website from the Swiss-Prot database previously. They searched for PTM vocabularies only in the FT field of each protein, found 431 PTM vocabularies and divided them into two distinct categories: ‘Putative dictionary’ and ‘Experimental dictionary’. Based on these two categories, we extracted related statistics of PTM terms and compared them with UniProt search engine (Table 1). The results indicated Phosphoserine, N6-(pyridoxal phosphate) lysine, and Phosphothreonine as the most frequent experimental PTM vocabularies. The frequencies of these vocabularies were 8337, 4871 and 3165, respectively. Phosphoserine, Phosphothreonine and N6-acetyllysine were also obtained as the most frequent putative experimental PTM vocabularies with frequencies 16 067, 7220 and 6923, respectively. It should be noted that the frequency of proteins with two non-experimental qualifiers ‘Probable’ and ‘Possible’ has been significantly reduced since 2011. This shows that the UniProtKB/Swiss-Prot information is rapidly updating and more proteins are reviewed.

**Table 1.** The top 10 ranked PTM terms based on Khoury *et al.* and PEIMAN software

	Putative		Experimental		Total	
	Khoury *et al.*	PEIMAN	Khoury *et al.*	PEIMAN	Khoury *et al.*	PEIMAN
1	N-linked glycosylation (98 732)	Phosphoserine (16 067)	Phosphoserine (30 795)	Phosphoserine (8337)	Phosphoprotein (108 222–39 733*)	Nucleotide-binding (97 643–117 591*)
2	Phosphoserine (39 478)	Phosphothreonine (7220)	Phosphothreonine (6031)	N6-(pyridoxal phosphate)lysine (4871)	N-linked glycosylation (104 966–437*)	Phosphoprotein (36 917–39 733*)
3	N6-acetyllysine (16 852)	N6-acetyllysine (6923)	N-linked glycosylation (5996)	Phosphothreonine (3165)	Acetylation (33 291–18 702*)	Disulfide bond (32 930–33 277*)
4	Phosphothreonine (10 291)	Phosphotyrosine (3684)	N6-acetyllysine (4929)	N-palmitoyl cysteine (1909)	Methylation (10 295–11 066*)	Glycoprotein (28 874–29 827*)
5	N6-(pyridoxal phosphate)lysine (6311)	N-acetylalanine (2986)	Glycyl lysine isopeptide (4919)	S-diacylglycerol cysteine (1909)	Palmitoylation (6069–1137*)	Phosphoserine (24 395–24 565*)
6	Phosphotyrosine (5808)	N-acetylmethionine (2456)	Phosphotyrosine (2176)	Phosphohistidine (1846)	Amidation (5548–3639*)	Acetylation (17 407–18 702*)
7	N6-succinyllysine (5397)	Glycyl lysine isopeptide (2121)	N-acetylalanine (1452)	Cysteine persulfide (1802)	Citrullination (4808–289*)	Phosphothreonine (10 385–10 513*)
8	Citrulline (4670)	Glycyl lysine isopeptide (Lys-Gly) (2032)	N6-succinyllysine (1380)	N6-acetyllysine (1773)	O-linked glycosylation (4104–343*)	Ubl conjugation (9182–9408*)
9	S-palmitoyl cysteine (3578)	N6-succinyllysine (2021)	O-linked glycosylation (1343)	N6-carboxylysine (1436)	Sulfation (3842–697*)	N6-acetyllysine (8693–8704*)
10	O-linked glycosylation (2684)	S-palmitoyl cysteine (1943)	Interchain with G-Cter in ubiquitin (1136)	N5-methylglutamine (1224)	Hydroxylation (3259–1669*)	Lipoprotein (8409–10 968*)

The starred numbers indicate the number of UniProt search engine hits. The small differences between PEIMAN database and UniProtKB are due to our strategy to find PTM terms in FT and KW fields only.

The Ontobee, a web-based software which is proposed by Xiang *et al.* (27) for biomedical ontologies, provides a DAG for PTM terms. This DAG contains four nodes at the second level containing: ‘protein modification categorized by amino acid modified’, ‘protein modification categorized by chemical process’, ‘protein modification categorized by isobaric sets’ and ‘uncategorized protein modification’. Although this design is useful in order to organize biological concepts related to PTMs, these terms are not compatible with UniProt databases. In this study, the most recently found vocabularies in UniProtKB were applied to construct a DAG for PTM in three levels. In comparison to Ontobee, PEIMAN proposed DAG for PTM vocabularies based on the relation between KW and FT terms.

Finally, Li *et al.* (16, 18) represented a curated, web- accessible PTM database called SysPTM in two different versions which systematically reviews the resources of PTM and provides four tools to predict functional analysis of PTMs including: PTMBlast, PTMPathway, PTMPhylog and PTMGO. The last module (PTMGO) has been allocated to perform PTM enrichment analysis. The new version of this software covers 50 PTM types with covering only 2031 species. PEIMAN software allows to perform PTM enrichment analysis for 511 different PTM types which are found among 8685 species across UniProtKB/Swiss-Prot. This feature makes the results of PTM enrichment significantly more accurate and reliable.

As proof of the concept, we accomplished PTM enrichment for various sets of protein targets of drugs using PEIMAN. The dataset was downloaded from DrugBank website. The enriched PTM types were consistent with their cellular location and biological function. As an example of proof, receptor targets which were located on cell membrane were highly enriched for glycoprotein sulfation. For targets of kinase inhibitors, phosphorylation and ubiquitination were observed. Metaloenzymes were shown to undergo proteolysis (Bonferroni-corrected *P*-value <0.05 was considered significant). Mapping such modifications in drug targets, complementarity with pharmacogenomics studies, helps understanding the underlying causes of variation in drug response among individuals.

## Conclusion

Among the studies regarding PTMs, a comprehensive database for PTM enrichment analysis with considering all available PTM terms was not considered yet. PEIMAN can be considered in enrichment analysis for a given protein list or to compare the PTM in two protein lists for matching and integration. It has also the capability of reporting the frequency of PTMs in a desired list of proteins. Since the database is regularly updated as the new version of UniProt is released, the results are in accordance with UniProt database. This study hopes to improve the approach in collecting PTMs annotation. The improvement was provided by searching in both KW and FT fields and tried to find more PTMs which are already available in UniProt/Swiss-Prot database. PEIMAN database has considered the most number of species and proteins and associated PTMs which was not considered yet.

## Supplementary Data

Supplementary data are available at *Database* Online.
